# Editorial: Looking at it from a different angle: Positive aspects and strengths associated with neurodevelopmental disorders

**DOI:** 10.3389/fpsyt.2022.1111086

**Published:** 2022-12-23

**Authors:** Martine Hoogman, Fengyu Zhang, Corina U. Greven

**Affiliations:** ^1^Departments of Psychiatry and Human Genetics, Donders Institute for Brain, Cognition and Behaviour, Radboud University Medical Center, Nijmegen, Netherlands; ^2^Global Clinical and Translational Research Institute, Bethesda, MD, United States; ^3^Department of Cognitive Neuroscience, Donders Institute for Brain, Cognition and Behaviour, Radboud University Medical Center, Nijmegen, Netherlands; ^4^Karakter Child and Adolescent Psychiatry University Centre, Nijmegen, Netherlands; ^5^Institute of Psychiatry, Psychology and Neuroscience, Social, Genetic and Developmental Psychiatry Centre, King's College London, London, United Kingdom

**Keywords:** autism, ADHD, neurodiversity, neurodevelopmental conditions, strengths

Attention Deficit/Hyperactivity Disorder (ADHD) and autism spectrum disorder (ASD) are common neurodevelopmental conditions. People with these conditions face different challenges, from symptom-related difficulties such as sustaining attention, to finishing their education and holding a job, and dealing with stigma. The social and economic costs of ADHD and ASD are tremendous, with the majority of costs due to productivity loss (e.g., work participation), special education costs and loss of wellbeing (e.g., years of healthy life lost) (1, 2). These conditions are highly heterogeneous; while some people have an IQ more than two standard deviations below the mean and/or strong functional impairments; others may have highly functional traits. In trying to better understand these disorders, research has so far mainly focussed on the deficits associated with these conditions. However, there is also evidence for positive aspects, strengths or excellence associated with these conditions. While there have been famous people with autism spectrum disorder who made breakthroughs in multiple fields in the human history, the scientific evidence on strengths in the broader group of individuals with these conditions is scarce. We think that approaching ADHD and ASD from a different angle, such as for example investigating strengths, will deliver new information that might boost our objective and complete understanding of these conditions.

We are proud to present this Research Topic “*Looking at it from a different perspective: positive aspects and strengths associated with neurodevelopmental conditions*.” It comprises of seven articles that have adopted a different outlook and therefore have generated novel information about ADHD and ASD.

Two studies aimed to compare ADHD and ASD on the specific cognitive functions of attention and creativity. Based on a community sample of ADHD (*n* = 346) and ASD (*n* = 131) children, Dupuis et al. found that autistic children had greater attentional strength than children with ADHD did. However, attentional strength was not associated with impairment and cognitive flexibility in ASD. In Stolte et al., different aspects of creativity (divergent, convergent thinking and creative achievements) were investigated in relation to ADHD and ASD symptoms in the general adult population (*n* = 470). They found divergent thinking to be positively correlated with the number of ADHD symptoms; this was confirmed in a different case-control sample. Also, creative achievements in the domain of “expression” (e.g., humor) were associated with ADHD. For ASD no obvious associations with creativity were found but the ASD subdomains of “imagination” and “social difficulties” generated questions for future research.

Previous studies on positive ADHD-related characteristics have largely focused on one particular strength, namely creativity. Creativity has been reported in individuals who had clinical or subclinical symptoms of psychiatric conditions such as ASD, ADHD, bipolar disorder, and schizophrenia, although its association is not very straightforward (3–7). Stolte et al. extended previous work by examining the association between sensory and sensorimotor gating and creativity or attentional difficulties among children. The gating abilities were measured with P50 suppression (*n* = 65) and Prepulse inhibition of the startle reflex (*n* = 37). Creativity was measured by creative thinking-drawing production and subcomponents of divergent creativity (in originality, fluence, and flexibility). They showed that children with a higher P50 testing amplitude tend to have a higher divergence of fluence and flexibility at a nominal significance level. Prepulse inhibition was not associated with either general creativity or its subcomponents. This study showed that although sensory- and sensorimotor gating and attentional difficulties are unrelated, creativity and quantitative measures of divergent thinking such as fluency and flexibility, are at least indirectly related to decreased sensory, but not sensorimotor gating. Also, lower sensorimotor gating seems to benefit tasks related to general creativity.

Schippers et al. aimed to extend the identification of strengths in ADHD to move beyond researching creativity alone. They performed a citizen science project, collaborating with the Dutch organization for people with ADHD, and collected data on self-reported positive characteristics of ADHD in 206 adults with ADHD. This resulted in a list of 116 positive aspects which were categorized into *Creativity, Being dynamic, Flexibility, Socio-affective skills*, and *Higher-order cognitive skills*. They concluded that awareness about strengths of ADHD might help individuals with ADHD and their environment to better cope with and accept their diagnosis. Awareness of strengths might also facilitate better choices for education or occupation. However, to incorporate the self-reported positive aspects in the understanding of ADHD, future research should focus on quantifying strengths in ADHD relative to non-ADHD samples, and investigating the link between these aspects and clinical characteristics of ADHD. A goal of this work is to implement such new knowledge in psychoeducation and find its way to society.

In their theoretical paper, Colzato et al. aim to move away from a unipolar view of cognitive control, in which optimal cognitive control is characterized by complete persistence on one goal while ignoring goal-irrelevant information. In the meta-cognitive control model, the authors suggest replacing this with a bipolar account of cognitive control. Here, variation in cognitive processing style is characterized by two poles: one of persistence—which corresponds to the original idea of cognitive control as described in the unipolar model—and one of flexibility—which is less selective and exclusive and facilitates switching between tasks, ideas and actions and is beneficial for tasks requiring integration of information with varying relevance. The authors conclude by outlining the relevance of their model to task performance in neurodevelopmental disorders such as autism and tic disorders.

Not only investigating strengths *per se* is a novel way of looking at neurodevelopmental conditions, also investigating positive environments can deliver important new information about neurodevelopmental conditions in the context of prevention and increased quality of life. Einziger et al. wrote a review about sensitivity to aspects of the home environment and parenting in the context of ADHD. These factors might moderate genetic liability and the negative effects of these environmental factors are well-established, but the opposite, the positive effect of high quality environments and positive parenting are under-investigated. The review contributes to increased insights for designing preventive interventions and for identifying those children that are especially sensitive and could benefit from such interventions; also recommendations for future work are given.

The work of Furukawa et al. is a timely reminder of the value of using input from children themselves for health-care but also for research. The authors investigated the wishes of 299 school-aged children with ADHD by means of using the Three Wishes task in which they could indicate what they wanted to change in their lives. Many wishes were related to immediate fulfillment but also to positive interpersonal relationships. This work highlighted the diversity and typicality of needs, desires and hopes of children with ADHD.

To conclude, the published articles in this collection highlight the diversity of strengths that are reported in the context of neurodevelopmental conditions to span personality, emotional, cognitive variables as well as responsivity to positive environmental stimulation. While almost all these areas are understudied in relation to neurodevelopmental conditions, we think that these aspects have the potential to change the prospects of individuals with such conditions for the better. We anticipate that the identification of these missing aspects will change the ways we use to approach neurodevelopmental conditions. It will have an impact on coping with the condition and will provide new perspectives for healthcare, as well as supporting improved quality of life.

## Author contributions

MH wrote the manuscript. FZ and CG critically reviewed the manuscript. All authors approved the final version.
